# Complete Genome Sequence of the *Pseudomonas aeruginosa* Bacteriophage phiIBB-PAA2

**DOI:** 10.1128/genomeA.01102-13

**Published:** 2014-02-06

**Authors:** Diana P. Pires, Andrew M. Kropinski, Joana Azeredo, Sanna Sillankorva

**Affiliations:** a Institute for Biotechnology and Bioengineering, Centre of Biological Engineering, Universidade do Minho, Campus de Gualtar, Braga, Portugal; b Public Health Agency of Canada, Laboratory for Foodborne Zoonoses, Guelph, ON, Canada; c Department of Molecular and Cellular Biology, University of Guelph, Guelph, ON, Canada

## Abstract

*Pseudomonas aeruginosa* phage phiIBB-PAA2 is a broad-host-range virus isolated from raw hospital sewage (Porto, Portugal). This phage has a terminally redundant (183 bp), 45,344-bp double-stranded DNA (dsDNA) genome encoding 66 coding sequences (CDSs) and 3 tRNAs. It belongs to the family *Podoviridae* and the genus *Luz24likevirus*.

## GENOME ANNOUNCEMENT

*Pseudomonas aeruginosa* is a significant opportunistic pathogen frequently associated with nosocomial infections. The infections caused by this bacterium are generally related to its ability to form biofilms on a variety of surfaces. These infections are difficult to treat due to the low susceptibility of the biofilms to antibiotics (1, 2).

The strictly lytic phage phiIBB-PAA2 was isolated from hospital wastewater in Porto, Portugal, using a reference strain (ATCC 10145) as the host. Our group previously showed that this phage has a broad lytic spectrum even against multidrug-resistant *P. aeruginosa* clinical strains (3).

The transmission electron microscopy (TEM) morphological characterization of phiIBB-PAA2 showed that it belongs to the family *Podoviridae*. The phage genome was sequenced using Roche/454-recommended procedures at the Plateforme d’analyses of the Institut de Biologie Intégrative et des Systèmes (Laval University, Québec, QC, Canada). Shotgun reads were assembled using the gsAssembler module of Newbler v 2.5.3. The potential coding sequences (CDSs) were initially annotated using myRAST (4). Each of the predicted proteins was screened against the National Center for Biotechnology Information (NCBI) protein databases, using BLASTP (5). Promoter sequences were predicted using PHIRE 1.0 (6) and BPROM (7), while predicted terminators were annotated using ARNold (8). tRNAscan-SE (9) was used for tRNA annotation.

The genome of the phage phiIBB-PAA2 consists of a linear double-stranded DNA (dsDNA) of 45,344 bp with a GC content of 52% and two 183-bp-long direct terminal repeats (DTRs) determined by Sanger DNA sequencing. The genome was scanned for CDSs, resulting in 66 predicted genes ranging from 141 bp to 3,168 bp, 45 of which are rightward oriented while the others are leftward oriented. The initiation codon of 89% of the genes is ATG, while 8% start with GTG and 3% with TTG. Based on the BLASTP analyses, only 25% of the proteins have assigned functions.

Our analyses further reveal that phage phiIBB-PAA2 has 8 host-dependent promoters, 3 terminators, and 3 tRNAs. The closest relative of phage phiIBB-PAA2 is *Pseudomonas* phage LUZ24 (10), with which it shares 83.2% nucleotide identity.

### Nucleotide sequence accession number.

The complete genome of the *P. aeruginosa* phage phiIBB-PAA2 has been deposited in GenBank under the accession number KF856712.
